# Induced membrane technique for the treatment of chronic hematogenous tibia osteomyelitis

**DOI:** 10.1186/s12891-017-1395-6

**Published:** 2017-01-23

**Authors:** Xiaohua Wang, Zhen Wang, Jingshu Fu, Ke Huang, Zhao Xie

**Affiliations:** National & Regional United Engineering Laboratory of Tissue Engineering, Department of Orthopaedics, Southwest Hospital, Third Military Medical University, Chongqing, 400038 People’s Republic of China

**Keywords:** Induced membrane, Two-stage surgery, Hematogenous osteomyelitis

## Abstract

**Background:**

Chronic hematogenous osteomyelitis often results from the improper treatment of acute hematogenous osteomyelitis. At present, there is lack of uniform standards for the treatment, and the clinical features of the disease are unclear. The purpose of this study was to explore the clinical efficacy and complications of chronic hematogenous tibia osteomyelitis treated with the induced membrane technique.

**Methods:**

A retrospective analysis of the chronic hematogenous tibia osteomyelitis patients in our department admitted from January 2013 to February 2014 and treated with the induced membrane two-stage surgical technique was performed. The defects were filled with antibiotic-loaded polymethyl methacrylate (PMMA) cement after radical debridement, and bone grafts were implanted to repair the defects after 6 to 8 weeks.

**Results:**

A total of 15 cases were admitted in this study, including 13 men and 2 women with a mean age of 34 years (6 to 51). The mean duration of bone infection was 142 months (3 to 361). All patients were cured with an average follow-up of 25 months (24 to 28). Radiographic bone union occurred in 5.3 months (3 to 8), and full weight bearing occurred in 6.7 months (4 to 10). No recurrence of infection was noted at the last follow-up. Two cases required repeated debridement before grafting due to recurrent infection. One patient had a small bone diameter due to insufficient grafting, and one patient had limitation of knee activity.

**Conclusions:**

The induced membrane technique for the treatment of chronic hematogenous tibia osteomyelitis is an effective and reliable method. Thorough debridement and wound closure at the first stage is essential for infection control as well as sufficient grafting at the second stage to ensure bone union.

## Background

Any form of inflammation involving bone tissue or marrow caused by a pathogenic organism is called osteomyelitis [1]. The condition mostly occurs at the metaphyseal of immunocompetent patients [2]. Hematogenous osteomyelitis is rare in developed countries but remains a serious problem in less developed regions. Chronic hematogenous osteomyelitis mainly occurs in long bones. In a rural African setting, tibia osteomyelitis was noted in 21.6% of these patients [3]. Adult hematogenous osteomyelitis was mostly persistent from childhood [4]. Blood flow is slower at the growth bone site, so the bacteria deposited cause acute osteomyelitis [5]. If not treated in time or with improper treatment, the condition will persist and become chronic osteomyelitis. Although great progress has made in the treatment of osteomyelitis, there is no standard for clinicians to use. According to our knowledge, few reports on the treatment of chronic hematogenous tibia osteomyelitis are available, and the characteristics of the disease are unclear. In recent years, Masquelet reported [6] a two-stage surgical method for the treatment bone defects, which is called induced membrane technique, and acquired great success. This technique is recognized and used in the treatment of osteomyelitis [7]. We reported the use of the induced membrane technique for the treatment of 32 cases post-traumatic osteomyelitis and obtained good clinical efficacy [8]. Here, we performed a retrospective analysis of 15 patients with chronic hematogenous tibia osteomyelitis treated with the induced membrane two-stage surgical technique to assess the clinical outcome and provide a reference for this disease.

## Methods

After approval by the Ethics Committee, we retrospectively analyzed chronic hematogenous tibia osteomyelitis patients in our department from January 2013 to February 2014. The inclusion criteria were (1) historical evidence of radiological and clinical bone infection at the tibia; (2) no historical evidence of open fracture or fracture internal fixation at the involved site; (3) local bone pain and swelling on examination, imaging procedures, microbiology and histopathology, and laboratory studies [8]. The gold standard for diagnosis of osteomyelitis is biopsy or culture from deep tissue [9] frozen sections confirmed to have more than five neutrophils per high-power field. Those with insufficient information or with Cierny-Mader C stage, which is inoperable, were excluded. We collected the patient's age, sex, Cierny-Mader host stages, duration of bone infection, culture, fixation and grafting type by patient record systems.

Seventeen cases were treated with this technique, and two were excluded due to incomplete information. Thus, a total of 15 patients were reviewed (Table 1), including 13 men and 2 women with a mean age of 34 years (6 to 51). The mean duration of bone infection was 142 months (3 to 361). Six (40%) cases had a history of collision, and 11 (73.3%) patients were younger than 20 years old at their initial onset. There were 6 cases in the proximal tibia, 7 cases in the middle tibia, and 2 in the distal tibia. No patient had diabetes, immune deficiency diseases or peripheral vascular diseases. Two cases were classified as Cierny-Mader type II, 8 cases as type III and 5 cases as type IV. Eleven cases had draining sinus tracts or local redness and swelling, and the remaining 4 cases had a history of osteomyelitis. The main clinical manifestations were long-term pain at the involved site, and radiological examination revealed bone destruction. Nine patients had bacterial cultures containing 11 strains, including 4 strains of *Staphylococcus aureus*, 2 *Proteus*, 1 *Enterobacter cloacae*, 1 *Micrococcus luteus*, 1 hemolytic *Staphylococcus*, 1 fecal *Enterococcus* 1 *Klebsiella oxytoca*.

**Table 1:** Patient demographics

Patient number/sex	Location	Cierny-Mader types	Skin ulcer	Duration of infection	Bacterium	Bone defect (cm3)	Fixation		Time to WB/S2 (Months)
							Istage	IIstage	
1/M	Proximal	III	YES	30Y	Enterobacter cloacae	75	external fixation	external fixation	6
2/M	Middle	IV	YES	16Y	Staphylococcus aureus	30	EP	EP	7
3/M	Middle	III	NO	7Y	Micrococcus luteus	45	external fixation	external fixation	7
4/M	Proximal	IV	NO	10Y	Not found	60	EP	EP	9
5/F	Middle	III	NO	7 M	Not found	25	EP	EP	4
6/M	Middle	III	NO	2Y	Not found	35	IP	IP	7
7/M	Proximal	III	NO	9 M	Not found	42	IP	IP	6
8/M	Distal	III	YES	3 M	Staphylococcus aureus	20	IP	IP	4
9/M	Proximal	II	NO	30Y	Staphylococcus hominis, Efaecium	84	None	None	7
10/M	Proximal	IV	YES	31Y	Proteus mirabilis	75	EP	Nail	10
11/M	Distal	II	YES	13 M	Staphylococcus aureus	42	None	None	6
12/F	Middle	III	NO	6 M	Not found	30	EP	Nail	6
13/M	Middle	IV	YES	28Y	Staphylococcus aureus	60	EP	Nail	9
14/M	Middle	IV	YES	2Y	Proteus mirabilis, Klebsiella	80	IP	Nail	8
15/M	Proximal	III	NO	18Y	Not found	40	IP	IP	5

WB/S2: No pain full-weight bearing after the second stage; EP and IP: External and internal fixation with plate and screws

All patients were treated with the induced membrane technique. The defects were filled with antibiotic-loaded PMMA cement after radical debridement at the first stage. Then, bone graft was implanted to rebuild bone defects at the second stage. The range of debridement was determined with preoperative X-ray, CT, MRI and bone scintigraphy examinations [10, 11]. The sinus was removed, and the sequestrum was cleaned. Surrounding necrosis tissue and scar tissue should be removed because it causes tension, decreases the rate of wound healing, and acts as foci for infection [12]. The sequestrum and surrounding tissues are used for culture and pathological examination. The bone ends are subject to grinding until signs of bleeding, and the medullary cavity is reamed. The wound is washed with hydrogen peroxide, dilute povidone-iodine and saline repeatedly. After effective fixation, the defects are filled with 40 g PMMA cement (Heraeus, Germany) mixed with 5 ~ 10 g vancomycin (Eli Lilly, Japan). The cement should be packed in the bone ends and not be smaller than the diameter of the tibia. If internal fixation is used, the cement should wrap the bone. Then, suction drainage tubes are inserted, and the wound is closed. A local or distant flap will be useful for high skin tension. If infection recurs, repeated debridement was performed until the infection was controlled. Intravenous injections of sensitive antibiotics are administered for two weeks postoperatively based on the drug sensitivity tests. If the culture is negative, a third generation cephalosporin (Ceftazidime, Hailin, China) is administered at a dose of 2 g per 12 h. Suction drainage is performed for 10 ~ 12 d. Weight-bearing should not be performed between the first and second stages.

Grafting was performed 6 to 8 weeks later, with a mean interval time of 50 days (44 to 105). The situation of infection control should be assessed before grafting, including the presentation of signs, such as redness, sinus and pus, or abnormal laboratory tests, such as erythrocyte sedimentation rate, CRP and white blood cell count. These indicators could reveal recurrent infection, which requires debridement until the infection is under control. The amount of the graft is estimated based on CT measurement before surgery [13]. The cement is removed, and the canals are reamed. The wound is washed. The graft is cut to 0.5 cm × 0.5 cm × 0.5 cm in size. The induced membrane is grafted and sutured. Prophylactic antibiotics are administered for 24 h, and suction drainage is applied for 10 ~ 12 days.

Patients underwent follow-up at 1, 2, 3, 6, 9, 12, 18, and 24 months. We assessed the time to bone healing, infection control, local pain, range of knee movement, lower limb edema, ability to walk and other complications. Gradual ambulation until callus was observed. Our clinical endpoints were 24 months after the second stage until infection control and the X-ray scan revealed bone union. CT scans were performed when X-ray did not clearly indicate bone union. We defined radiographic healing as bridging callus on three of four cortices and clinical healing as pain-free full weight bearing [13].

## Results

The average volume of bone defects was 51.3 cm^3^ (20 to 84 cm^3^) after debridement. Three patients were implanted with autografts, 6 with autograft plus variable proportion of allograft and 5 with allograft for more than 25% of the total volume. Five children and one adult were implanted with allograft only. At the first stage, external fixations were used in 8 cases, 5 cases with internal fixation, five cases were replaced with nail at the second stage, and 2 cases with no fixation.

**Fig. 1:**
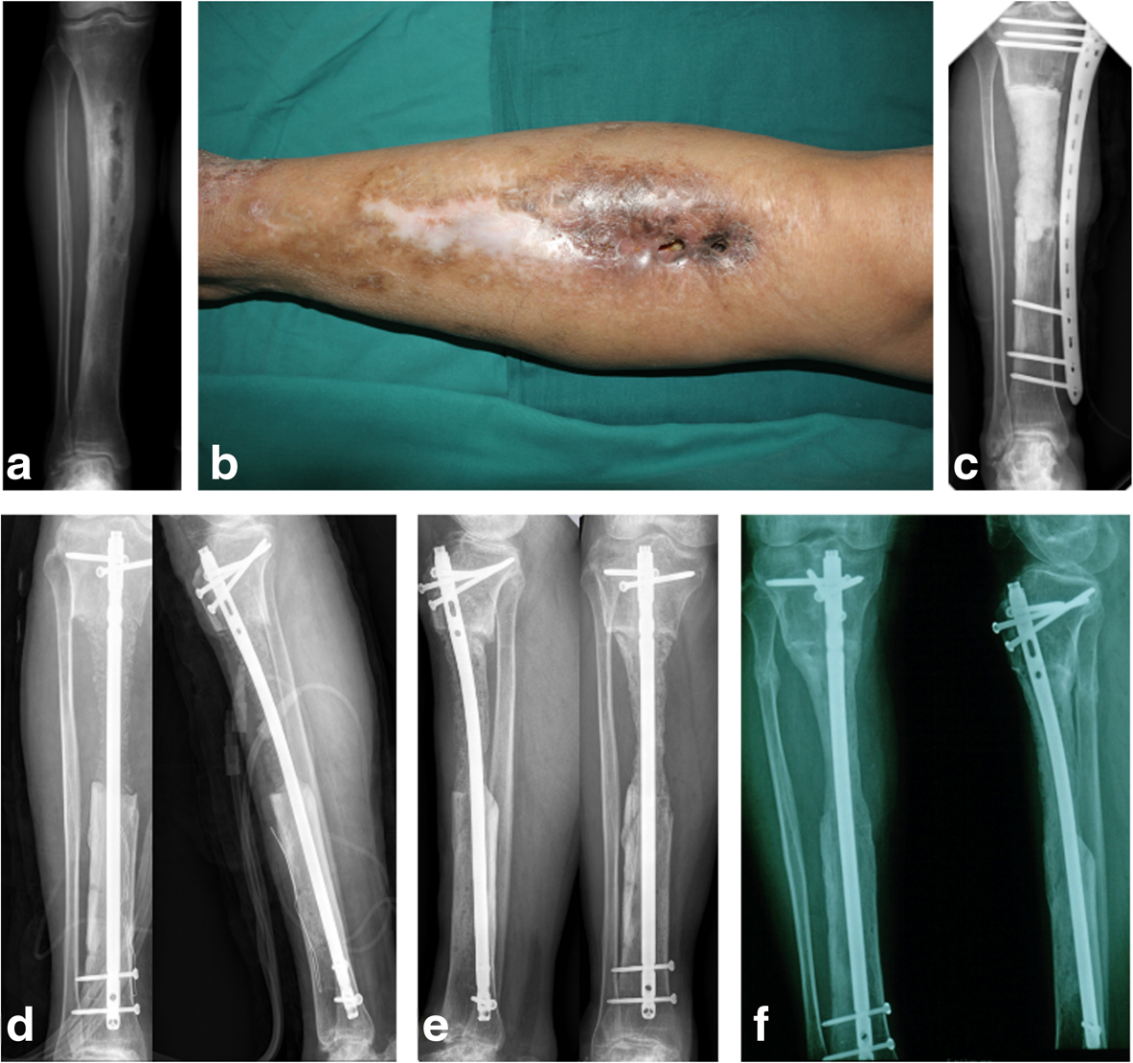
Case 10, 50 Y, repeated swelling and skin ulcer of the right proximal tibia for more than 30 years. *Proteus mirabilis* was identified. **a** and **b**: Sinuses were observed, and X-ray revealed bone destruction; **c**: Complete resection of the lesions and implantation with PMMA cement after the second stage; **d**: Grafting (autograft 60 ml + allograft 15 ml) eight weeks later; **e**: X-ray revealed bone union at 6 months; **f**: 20 months after the second stage, bone union with a small diameter was noted

**Fig. 2:**
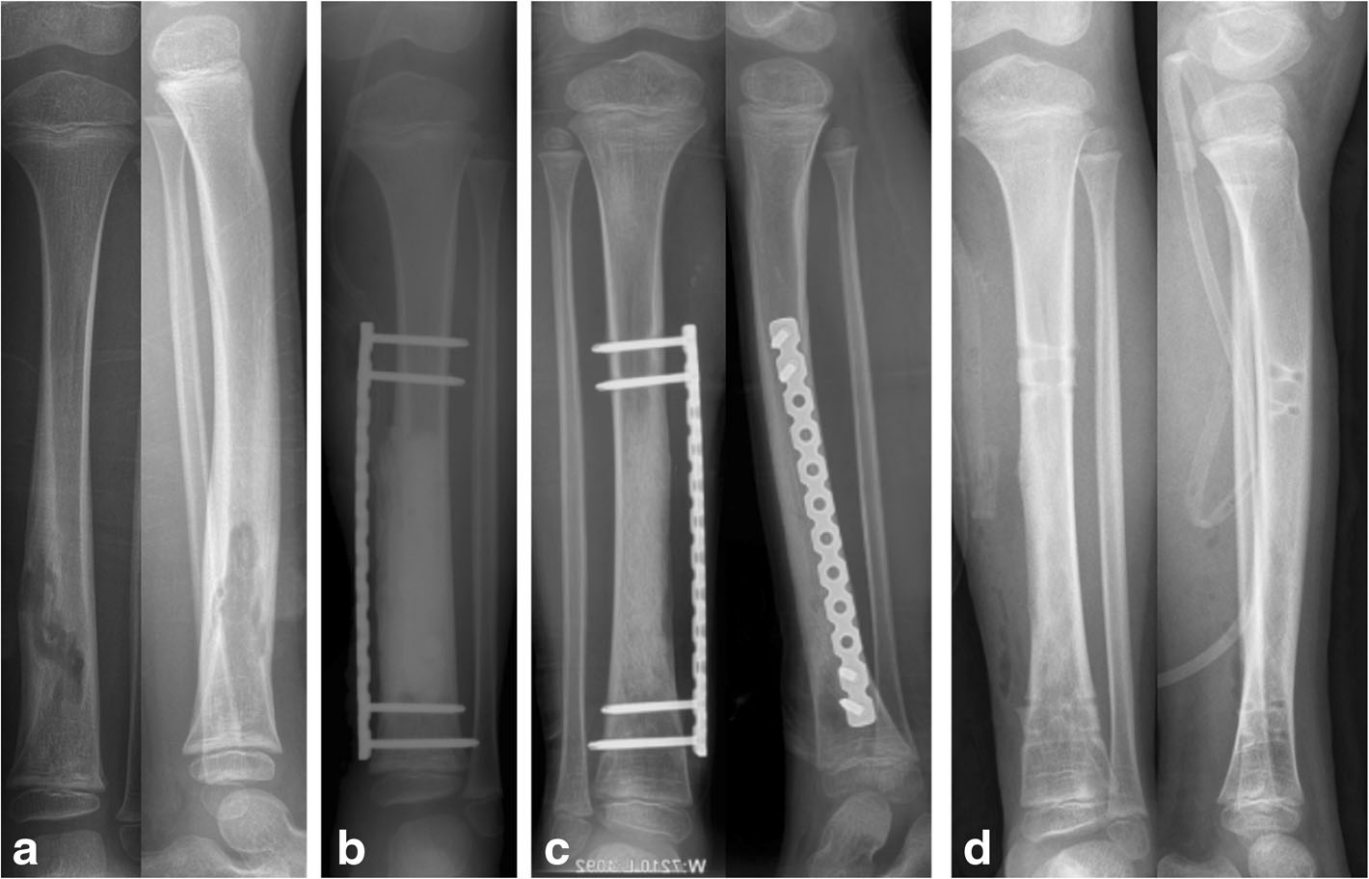
Case 8, 6 Y, Redness and painful left tibia for 3 M. The patient had a history of a fall 2 weeks before the illness; however, fracture and skin breakdown were not noted. Bone reconstruction was performed with an allograft. **a**: X-ray revealed bone destruction of the distal tibia; **b**: Implanted with PMMA cement and fixed with plate after debridement; **c**: X-ray revealed bone union 3 months after grafting; **d**: The bone defects were completely healed, and fixation was removed after 18 months

All the patients achieved bone union within the mean follow-up of 25 months (24 to 28), and no infection recurrence was noted. The mean radiographic bone union occurred in 5.3 months (3 to 8), and painless weight bearing was noted at 6.7 months (4 to 10). A comparison of the bone union time of three groups (allograft, allograft + autograft and autograft) did not reveal statistically significant differences (Table 2). Two patients required repeated debridement before grafting due to recurrent infection. Two cases with pin tract infection were noted when external fixation was used. Infection was controlled after the removal of the screw and topical sterilization. Four cases had dull pain or edema of the leg after activity. We recommended the use of symptomatic treatment and elastic stockings to reduce edema. Four cases had donor site pain and discomfort. One case had a small tibia diameter at the defect site (Fig. 1). One case had limitation of knee limb activity (0–85°). After an average of 25 months of follow-up, we observed no infection recurrence, and no lasting damage to bone was noted in all patients. Typical cases are presented in Fig. 2.

**Table 2:** Patients with different grafting

Graft type	Cases	Average age	Average bone defect (cm3)	Time to BU/S2 (Months)	Time to WB/S2 (Months)
Autograft	3	46.7Y	38.3	5.0	6.4
Autograft + Allograft	6	42.2Y	67.3	6.3	7.8
Allograft	6	19Y	37.3	4.5	5.8

BU and WB/S2:Radiographic bone union and no pain full-weight bearing after the second stage

## Discussion

Chronic osteomyelitis is a serious problem for orthopedists given the lack of a generally accepted method for treatment. Classic techniques include Ilizarov technique, one stage bone grafting and vascularized fibula graft. These techniques are often associated with high complications or long-term recurrence rates [14–16]. Some reports have described the use of the induced membrane technique in the reconstruction of long bone defects, but this technique has not been specifically studied in the treatment of osteomyelitis [4]. We applied the induced membrane technique for the treatment of chronic hematogenous tibia osteomyelitis. The process involves a series of measures, including radical debridement removal of necrotic tissue, elimination of dead space, drainage, skin covering and stable fixation for infection control. Then, a membrane is formed by implantation of a PMMA cement, and grafting the induced membrane approximately 8 weeks later. The membrane rapidly promotes bone defect repair [8].

Hematogenous osteomyelitis mostly occurs at the metaphyseal of children and less frequently in immunocompetent adults [2]. Local injury is an important factor for hematogenous osteomyelitis [17, 18]. Our results revealed that 6 (40%) patients had a history of collision at the involved site. Eleven (73.3%) patients were younger than 20 years old at their first outbreak, and these results were similar to a previous study. However, four immunocompetent adults with an initial outbreak were included. Clinical symptoms of chronic osteomyelitis include blurry vision, dull pain, chills and fever [10]. *Staphylococcus aureus* is the most common pathogen for bone and joint hematogenous osteomyelitis [19, 20], *Staphylococcus aureus*, *Streptococcus* and anaerobic bacteria accounted for 80% of all pathogens [21]. However, the proportion of bacteria may be altered with the course of the migration and surgical procedures. Our results showed that 9 patients were positive for 11 strains of bacteria. *Staphylococcus aureus* accounted for 26.7% (4 strains) of bacteria strains. Bacteria obtained from sinus secretions may not be reliable because the pathogens are often inconsistent with the deep tissue [10, 22]. Our positive bacterial rate was only 60% (9/15) for 5-day cultures. Sheehy SH et al. [23] reported a 64% positive rate with a 7-day culture, so they suggested prolonging the incubation time to 14 days.

Antibiotic therapy alone can acquire good clinical efficacy for acute hematogenous osteomyelitis, but chronic osteomyelitis often requires surgery due to the presence of a sequestrum [24]. Systemic antibiotics for osteomyelitis are generally administered for 4 to 6 weeks [25, 26]. However, this value is only based on empirical data. No study has demonstrated that 4 to 6 weeks of antibiotics can be more effective [10, 27]. Salgado reported the use of muscle versus non-muscle flaps for the treatment of chronic tibia osteomyelitis in animals, antibiotics for 5 days postoperatively, and no recurrence of infection within 1-year follow-up [28]. Knopp [29] reported 47 cases of chronic osteomyelitis, and intravenous antibiotics were administered for 3 to 5 days with an infection control rate of 85%. We used intravenous antibiotic therapy for approximately 2 weeks after the operation. No oral antibiotics were used given that radical debridement can disrupt bacterial biofilm formation, reduce bacterial load, improve the local blood supply, and enhance the efficiency of antibiotics. All of these measures prevent the further formation of bacterial biofilm and reduce the incidence of systemic adverse reactions [30]. If the surgeon is confident of thorough debridement, a shorter antibiotic time can be selected. If the skin and soft tissue conditions permit, we can also choose internal fixation, such as open fracture (contaminated wound), but the surface of the plate should be wrapped with bone cement. Bhaskar Borgohain [31] reported on an 8-year-old child. In this case, sequestrum disappeared with intravenous antibiotics and supportive treatment for 8 months without surgical intervention, but the shorter follow-up may exclude long-term infection recurrence.

The classic technique for the treatment of chronic osteomyelitis is often accompanied by complications. Muscle flap to fill the cavity is often applied, but it may affect the aesthetic and limit the function of donor site [28, 32]. Complications for treating hematogenous osteomyelitis include myositis, soft tissue abscess, fasciitis or blood borne complications, such as DVT [20]. Our results indicated that the complications mainly included leg edema, donor site pain and pin-track infection when external fixation was applied. Knee flexion is a limitation for proximal tibia osteomyelitis. One patient exhibited a small diameter of the tibia because the grafting affected wound closure. We reduced the volume of graft, so we recommend using a larger amount of cement compared with the corresponding diameter of the tibia at the first stage. Tibia osteomyelitis is often associated with skin defects. If large skin tension is noted after suturing, adjacent flap or skin graft can be necessary. Autograft is still the main source of the bone for this technique. Numerous studies indicated that greater than 25% bone substitute should not be added [33, 34]. Eleven (5 Autograft + Allograft and 6 Allograft) cases in our study were implanted with greater than 25% of the total volume of the allograft given that the PMMA cement induced the formation of a membrane, which is conducive to rapid healing of bone defects. We used a stem cell enrichment device to mix the autologous bone marrow with the allograft. Most patients had a partial bone defect with an increased repair ability. Children may be able to generate a periosteal membrane faster than adults [7], but their bone healing time was not significantly reduced in this study. This result could possibly be attributed to the fact that a greater proportion of the allograft was used for our skeletally immature patients. Formerly, we reported the induced membrane for the treatment of posttraumatic osteomyelitis [8]. When comparing the current results with our previous study on the application of the induced membrane technique of post-traumatic osteomyelitis, we found similar clinical efficacy and complications.

## Conclusions

The induced membrane two-stage surgical strategy for the treatment of chronic hematogenous tibia osteomyelitis is a clinically stable and reliable method. Thorough debridement and wound closure at the first stage, adequate grafting at the second stage and early functional exercises are necessary to achieve good clinical efficacy.

## Abbreviations

CRP: C-reactive protein
CT: Computed tomography
DVT: Deep vein thrombosis
MRI: Magnetic resonance imaging
PMMA: Polymethyl methacrylate.

## References

[1] Agarwal A, Aggarwal AN (2016). Bone and Joint Infections in Children: Acute Hematogenous Osteomyelitis. Indian J Pediatr.

[2] Thein R, Tenenbaum S, Chechick O (2013). Delay in diagnosis of femoral hematogenous osteomyelitis in adults: an elusive disease with poor outcome. Isr Med Assoc J.

[3] Ibingira CB (2003). Chronic osteomyelitis in a Ugandan rural setting. East Afr Med J.

[4] Sanders J, Mauffrey C (2013). Long bone osteomyelitis in adults: fundamental concepts and current techniques. Orthopedics.

[5] Shiels SM, Bedigrew KM, Wenke JC (2015). Development of a hematogenous implant-related infection in a rat model. BMC Musculoskelet Disord.

[6] Masquelet AC, Fitoussi F, Begue T (2000). Reconstruction of the long bones by the induced membrane and spongy autograft. Annales de chirurgie plastique et esthetique.

[7] Canavese F, Corradin M, Khan A, et al. Successful treatment of chronic osteomyelitis in children with debridement, antibiotic-laden cement spacer and bone graft substitute. Eur J Orthop Surg Traumatol. 2016:1-8. 10.1007/s00590-016-1859-7 27644427

[8] Wang X, Luo F, Huang K (2016). Induced membrane technique for the treatment of bone defects due to post-traumatic osteomyelitis. Bone Joint Res.

[9] Mouzopoulos G, Kanakaris NK, Kontakis G (2011). Management of bone infections in adults: the surgeon's and microbiologist's perspectives. Injury.

[10] Haidar R, Der Boghossian A, Atiyeh B (2010). Duration of post-surgical antibiotics in chronic osteomyelitis: empiric or evidence-based?. Int J Infect Dis.

[11] Tetsworth K, Cierny G, 3rd. Osteomyelitis debridement techniques. Clin Orthop Relat Res. 1999;360(360):87–96. 10.1097/00003086-199903000-00011 10101313

[12] Parsons B, Strauss E (2004). Surgical management of chronic osteomyelitis. Am J Surg.

[13] Stafford PR, Norris BL (2010). Reamer-irrigator-aspirator bone graft and bi Masquelet technique for segmental bone defect nonunions: a review of 25 cases. Injury.

[14] Dendrinos GK, Kontos S, Lyritsis E (1995). Use of the Ilizarov technique for treatment of non-union of the tibia associated with infection. J Bone Joint Surg Am.

[15] Gao YS, Ai ZS, Yu XW (2012). Free vascularised fibular grafting combined with a locking plate for massive bone defects in the lower limbs: a retrospective analysis of fibular hypertrophy in 18 cases. Injury.

[16] Giannoudis PV, Faour O, Goff T (2011). Masquelet technique for the treatment of bone defects: tips-tricks and future directions. Injury.

[17] Hwang HJ, Jeong WK, Lee DH, et al. Acute Primary Hematogenous Osteomyelitis in the Epiphysis of the Distal Tibia: A Case Report With Review of the Literature. J Foot Ankle Surg. 2016;55(3):600-604. 10.1053/j.jfas.2016.01.003 26878809

[18] Manche E, Rombouts-Godin V, Rombouts JJ (1991). Acute hematogenous osteomyelitis due to ordinary germs in children with closed injuries. Study of a series of 44 cases. Acta Orthop Belg.

[19] Agrawal R, Sharma D, Dhiman P (2015). Clinical and haematological predictors of acute hematogenous Methicillin Resistant Staphylococcus aureus (MRSA) osteomyelitis & septic arthritis. J Orthop.

[20] Ratnayake K, Davis AJ, Brown L (2015). Pediatric acute osteomyelitis in the postvaccine, methicillin-resistant Staphylococcus aureus era. Am J Emerg Med.

[21] Osman AE, Mubasher M, ElSheikh NE (2015). Investigation of polymorphisms in anti-inflammatory cytokine genes in hematogenous osteomyelitis. Genet Mol Res.

[22] Perry CR, Pearson RL, Miller GA (1991). Accuracy of cultures of material from swabbing of the superficial aspect of the wound and needle biopsy in the preoperative assessment of osteomyelitis. J Bone Joint Surg Am.

[23] Sheehy SH, Atkins BA, Bejon P (2010). The microbiology of chronic osteomyelitis: prevalence of resistance to common empirical anti-microbial regimens. J Infect.

[24] Rao N, Ziran BH, Lipsky BA (2011). Treating osteomyelitis: antibiotics and surgery. Plast Reconstr Surg.

[25] Lazzarini L, Mader JT, Calhoun JH (2004). Osteomyelitis in long bones. J Bone Joint Surg Am.

[26] Mader JT, Shirtliff ME, Bergquist SC (1999). Antimicrobial treatment of chronic osteomyelitis. Clin Orthop Relat Res.

[27] Shuford JA, Steckelberg JM (2003). Role of oral antimicrobial therapy in the management of osteomyelitis. Curr Opin Infect Dis.

[28] Salgado CJ, Mardini S, Jamali AA (2006). Muscle versus nonmuscle flaps in the reconstruction of chronic osteomyelitis defects. Plast Reconstr Surg.

[29] Knopp W, Kiztan T, Muhr G (1987). Soft tissue covers in chronic osteitis. Handchir Mikrochir Plast Chir.

[30] Henry SL, Seligson D, Mangino P (1991). Antibiotic-impregnated beads. Part I: Bead implantation versus systemic therapy. Orthop Rev.

[31] Bhaskar Borgohain NB, Tashi Khonglah. Complete incorporation of long diaphyseal sequestrum without surgical intervention in chronic hematogenous osteomyelitis of tibia in an immunocompetent child. Adv Biomed Res. 2014; 3(1):95. 10.4103/2277-9175.129365 PMC4007322 24800184

[32] Rubino C, Figus A, Mazzocchi M (2009). The propeller flap for chronic osteomyelitis of the lower extremities: a case report. Journal of plastic, reconstructive & aesthetic surgery. JPRAS.

[33] Masquelet AC (2003). Muscle reconstruction in reconstructive surgery: soft tissue repair and long bone reconstruction. Langenbeck's archives of surgery / Deutsche Gesellschaft fur Chirurgie.

[34] Masquelet AC, Begue T (2010). The concept of induced membrane for reconstruction of long bone defects. Orthop Clin North Am.

